# Quality of life and associated factors of heroin‐dependent patients receiving methadone and buprenorphine maintenance treatment

**DOI:** 10.1002/npr2.12402

**Published:** 2023-12-13

**Authors:** Hu‐Ming Chang, Ming‐Chyi Huang, Su‐Chen Fang, Shih‐Ku Lin

**Affiliations:** ^1^ Department of Addiction Sciences, Taipei City Psychiatric Center Taipei City Hospital Taipei Taiwan; ^2^ School of Medicine, College of Medicine Taipei Medical University Taipei Taiwan; ^3^ Department of Nursing Mackay Medical College Taipei Taiwan; ^4^ Department of General Psychiatry Chang Gung Memorial Hospital Taoyuan Taiwan

**Keywords:** buprenorphine, heroin dependence, medications to treat opioid use disorder, methadone, quality of life

## Abstract

**Aim:**

Although studies in Western countries have investigated the quality of life (QoL) of heroin users, limited research on this topic has been conducted in Asia. The present study assessed QoL in patients with heroin dependence receiving medications to treat opioid use disorder.

**Methods:**

We performed a cross‐sectional study of patients with heroin dependence receiving methadone and buprenorphine treatment. The demographic and substance use variables of patients receiving methadone and buprenorphine were compared. The Chinese Health Questionnaire (CHQ‐12), Obsessive Compulsive Drug Use Scale (OCDUS), and World Health Organization Quality of Life Short Form Taiwan version (WHOQOL‐BREF‐T) were administered to measure patient mental health problems, addiction severity, and QoL, respectively. Multivariate regression was used to identify the factors associated with QoL.

**Results:**

A total of 149 patients receiving methadone and 31 receiving buprenorphine completed the questionnaires. Individuals in the buprenorphine group were more likely to be married (*p =* 0.024) or employed (*p =* 0.024), have a higher educational level (*p =* 0.013), have lower drug craving (OCDUS: *p =* 0.035), or have higher QoL (WHOQOL‐BREF‐T: *p =* 0.004) than those in the methadone group. After adjustment for other variables, employment was positively associated with the physical, psychological, and environmental domains of QoL. Receiving buprenorphine treatment (*p =* 0.032) and longer treatment duration (*p =* 0.016) were associated with higher psychological QoL.

**Conclusion:**

Several factors were associated with QoL in patients with heroin dependence. Some measures may improve their QoL, such as reducing employment barriers, improving treatment adherence, or increasing accessibility to buprenorphine treatment.

## Abstract 2

Buprenorphine treatment, longer treatment duration, and being employed were associated with better psychological wellbeing in heroin‐dependent patients. Permitting eligible patients to take methadone home or reducing the cost of buprenorphine to promote its use should be considered to improve patients' life quality.

## Graphical abstract

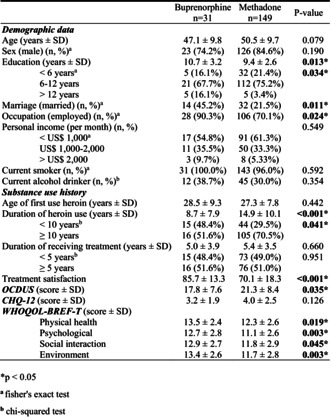

## 1 INTRODUCTION

Heroin is a highly addictive opioid and is associated with a considerable global burden.^1^ Heroin dependence is associated with the increased risks of death from drug overdose, transmission of blood‐borne diseases, and mental health problems. Heroin users have a higher risk of premature death than do nonusers. A meta‐analysis of 58 prospective cohort studies revealed a pooled standardized mortality ratio of 14.66 for opioid users.^2^ In a cohort of people with opioid use disorder in California, the estimated average years of life lost was 18.3 years compared with that of the general population.^3^ The introduction of medications to treat opioid use disorder (MOUD) has been effective in reducing drug use, improving health outcomes, and reducing the risk of drug overdose and infectious disease transmission. Using data from the National Mortality Registry in Taiwan, Chang et al. identified that receiving MOUD saves 7.8 years of potential years of life lost compared with not receiving MOUD.^4^ In the subsequent analysis, they demonstrated that receiving MOUD saves 9.7 quality‐adjusted life years compared with not receiving MOUD, which implies that MOUD improves the quality of life (QoL) of people with opioid use disorder.^5^

Traditionally, life expectancy is considered a key indicator of health. As the life expectancy of heroin users has increased, there has been a shift in the research focus to the measurement of health as a multidimensional concept including QoL. Although a consensus has not been reached on the definition of QoL, most studies define QoL as a multidimensional construct with an emphasis on the subjective evaluation of one's life circumstances.^6^ QoL is a key indicator of treatment outcomes and can guide policymaking. QoL is influenced by various factors, which can be modified by social services.

Studies have consistently demonstrated that individuals with heroin use disorder have lower QoL than the general population or individuals with other mental health problems.^7^, ^8^, ^9^ Several factors are associated with the lower QoL among individuals with heroin dependence, such as having a comorbid chronic disorder, legal problems, or history of imprisonment.^10^, ^11^, ^12^ In previous research, MOUD has the potential to enhance QoL for people with opioid use disorder. Some studies suggest that its effect on QoL is most prominent at the beginning of treatment,^13^ while others studies showed that the improvement in QoL can persist for a long time.^14^ Studies have identified that both methadone and buprenorphine treatment improve QoL in patients with opioid dependence, although inconsistent results have been obtained in comparative studies.^15^, ^16^, ^17^, ^18^

The factors associated with QoL may differ across cultures.^19^ In a cross‐cultural study, the association between levels of empowering knowledge and postoperative QoL in patients with osteoarthritis receiving surgery was found in Finland, Iceland, and Sweden, but not in Spain or Greece,^20^ which was partly related to participants' inherent knowledge of health. Most Asian countries have collectivist cultures, such as Korea, Japan, and China, where individuals place greater emphasis on fulfilling social roles and responsibilities, including maintaining employment.^21^ In addition, because of differing attitudes and regulations regarding illicit drug use across countries, levels of stigmatization differ across countries.^22^ Drug‐related policies also differ; although take‐home methadone is available in the United States, the United Kingdom, Australia, and Canada, it is not available in most Asian countries.

Although considerable research has investigated QoL in people with opioid use disorder, such studies in Asian countries are lacking. The Taiwanese government launched a harm reduction program in 2006 to address high human immunodeficiency virus (HIV) prevalence among people who inject drugs; this program included methadone treatment, needle and syringe, and educational programs.^23^ Heroin was the most popular illicit drug in Taiwan before 2008, according to reports from the Taiwan Food and Drug Administration. Starting in 2009, there was a decreasing trend in the prevalence of heroin use, which might be related to the implementation of the national harm reduction program in 2006.^24^ While methadone was introduced in the harm reduction program implemented by the Taiwanese government in 2006, buprenorphine then became available as MOUD in 2009.^25^ Buprenorphine was previously prescribed as an analgesic in sublingual or intravenous form. It was only after 2009 that it was formulated into sublingual tablets with naloxone for use in MOUD in Taiwan. In the present study, we recruited patients receiving MOUD for more than 3 months in Taiwan. The primary aim of the study was to ascertain the factors associated with QoL among patients with heroin dependence receiving MOUD. We also compared the clinical characteristics of patients receiving methadone and buprenorphine.

## 2 METHODS

### 2.1 Study population and eligibility

This cross‐sectional study was conducted at Taipei City Hospital between June 2017 and January 2018. This study recruited outpatients with heroin dependence receiving an MOUD program. Patients were eligible for the study if they met the following criteria: they (1) were older than 20 years; (2) used heroin as the main drug of abuse and opioid dependence had been diagnosed according to the *Diagnostic and Statistical Manual of Mental Disorders*, *Fourth Edition, Text Revision* criteria; and (3) had been either treated with buprenorphine or methadone for more than 3 months, considering that 3 months is defined as early remission of substance use disorder in the DSM‐5. Patients receiving involuntary treatment because of violating the Controlled Drugs Act were excluded. Patients who exhibited insufficient mental capacity to complete the questionnaires were excluded. This study was approved by the Ethics Committee of Taipei City Hospital (TCHIRB‐10602113) and conformed to the provisions of the Declaration of Helsinki.

### 2.2 Maintenance treatment program

At Taipei City Hospital, physicians administer two types of MOUD, namely methadone and buprenorphine; both treatments are funded by the government. The methadone treatment program is fully funded by the government, and the fee for treatment is approximately US$25 per patient per month, including the physician service fee and laboratory examinations. By contrast, the buprenorphine treatment program is partially funded and has a treatment fee of US$100 to 250 per patient per month depending on the dose of buprenorphine.

Whether patients receive methadone or buprenorphine is determined according to the physician's clinical assessment and the patient's preference. Only those who received involuntary treatment because of violating the Controlled Drugs Act were required to receive methadone treatment. A flexible dosing regimen is used, in which the doses are adjusted based on the severity of withdrawal symptoms. During the initial visit, the patient receives a complete physical examination, including chest X‐ray, electrocardiography, and blood tests for HIV, syphilis, and hepatitis B and C. At the first visit, 10–40 mg methadone or 2–8 mg sublingual buprenorphine is given. In subsequent visits, the dose is increased depending on the patient's condition. The patient visits a psychiatrist for drug refills every 2–4 weeks. Random urine screening tests are conducted to detect heroin use during each visit. Patients with positive urine screen tests are interviewed to assess the necessity of dose adjustment.

### 2.3 Study procedure and instruments

A trained research assistant screened outpatients participating in the MOUD program for more than 3 months. Participants were informed of the anonymous and voluntary nature of study participation and provided their written informed consent. Patients who completed the questionnaires distributed by the research assistant were compensated with US$5.

Demographic and substance use profiles were collected. Substance use history included the age at first use of heroin, duration of heroin use, and duration of maintenance treatment. Subjective satisfaction regarding maintenance treatment was assessed using the visual analog scale, with score ranging from 0 to 100. QoL, mental health condition, and drug craving severity were assessed using the World Health Organization Quality of Life Short Form Taiwan version (WHOQOL‐BREF‐T), Chinese Health Questionnaire (CHQ‐12), and Obsessive Compulsive Drug Use Scale (OCDUS), respectively.

#### 2.3.1 World Health Organization quality of life short form Taiwan version (WHOQOL‐BREF‐T)

The WHOQOL‐BREF is the brief version of the original long form of the WHOQOL questionnaire (WHOQOL‐100) and contains 28 items rated using a 5‐point scale; this questionnaire measures QoL in the following four domains: physical health, psychological health, social relationships, and environmental health. Higher WHOQOL‐BREF scores indicate higher QoL. The WHOQOL‐BREF exhibited satisfactory measurement characteristics in a Norwegian national sample.^26^ The WHOQOL‐BREF‐T was developed in 1999 and included two additional culture‐specific items (being respected and eating) to the social and environmental domains. The WHOQOL‐BREF‐T is a validated questionnaire with internal consistency (Cronbach's *α*) coefficients of 0.70–0.77 and content validity coefficients of 0.53–0.78 for item–domain correlations.^27^ The psychometric properties of the WHOQOL‐BREF‐T have been validated in various population, including adolescents,^28^ the elderly,^29^ cancer patients,^30^ and patients on hemodialysis.^31^ The WHOQOL‐BREF‐T for treatment outcome measurement in patients with heroin dependence has been validated.^32^

#### 2.3.2 Chinese health questionnaire (CHQ‐12)

The Chinese Health Questionnaire (CHQ) was originated from the General Health Questionnaire, which was developed by Goldberg in the 1970s for screening mental health problems in the general population.^33^ Given the cultural differences between Western and Chinese societies, the CHQ not only addresses direct mental health expression but also encompasses two novel aspects relevant to Chinese culture, including somatization (the physical manifestations of psychological problems) and family relationships. The questionnaire was originally developed as a 60‐item instrument, but the 12‐item version is as valid as the longer version.^34^ While some studies showed the CHQ‐12 is a unidimensional questionnaire,^34^ others have suggested that the CHQ‐12 encompasses three factors, namely somatic symptoms, anxiety, and depression/poor family relationships.^35^

The CHQ‐12 contains 12 items rated on a 4‐point scale, and higher scores indicate poorer mental health. In the present study, we applied the bimodal scoring method (0‐0‐1‐1), with a total score of 0–12. The validity of the CHQ‐12 has been demonstrated clinically, with Cronbach's α coefficients of 0.83 in primary healthcare settings and 0.84 in communities.^34^

#### 2.3.3 Obsessive compulsive drug use scale (OCDUS)

The OCDUS was modified from the Obsessive Compulsive Drinking Scale, which originated from the Yale–Brown Obsessive Compulsive Scale.^36^ The OCDUS contains 12 items rated using a 5‐point scale, with a total score of 12–60, and the scale measures heroin cravings in the preceding week. Higher scores indicate stronger drug craving. The OCDUS comprises three factors: thoughts concerning heroin and interference, desire and control, and resistance to thoughts and intention. Adequate reliability has been found for these domains, with Cronbach's *α* values of 0.90, 0.84, and 0.91.^37^

### 2.4 Data analysis

We conducted a descriptive analysis to compare the characteristics of patients receiving methadone and buprenorphine. Continuous variables were analyzed using Student's *t*‐test, and categorical variables were analyzed using the chi‐square or Fisher's exact test.

Univariate linear regression was used to examine the associations of methadone and buprenorphine treatment with demographic profiles, substance use profiles, general mental health condition (i.e., CHQ‐12 score), drug craving severity (i.e., OCDUS score), and QoL (i.e., each domain score of the WHOQOL‐BREF‐T). In univariate linear regression, demographic profiles, substance use profiles, general mental health condition, and drug craving severity were included as independent variables, and each domain score of the WHOQOL‐BREF‐T was a dependent variable.

Multivariate regression was used to identify potential factors independently associated with QoL (i.e., each domain score of the WHOQOL‐BREF‐T). A total of 14 potential factors were considered. These factors were age, sex, years of education, marital status, occupation status, personal income, current smoking habits, current alcohol drinking habits, treatment with methadone and buprenorphine, age at first heroin use, duration of heroin use, duration of treatment, general mental health condition (i.e., CHQ‐12 score), and drug craving severity (i.e., OCDUS score). Collinearity was assessed based on the statistical factor of tolerance and the variance inflation factor. Personal income was entered as a dummy variable.

A *p*‐value of <0.05 indicated significance. All statistical analyses were conducted using SPSS version 20 (SPSS, Chicago, Illinois, USA).

## 3 RESULTS

A total of 204 eligible patients with opioid dependence were invited to participate in the study, and 180 completed the questionnaires, comprising 31 patients receiving buprenorphine and 149 receiving methadone. Their demographic profiles, substance use history, and questionnaire scores are listed in Table 1. The age and sex distribution did not differ significantly between the buprenorphine and methadone groups. Compared with participants in the methadone group, more participants in the buprenorphine group had a higher educational level (*p =* 0.013), had been married (*p =* 0.011), and maintained a job (*p =* 0.024). Participants in the buprenorphine group had a shorter duration of heroin use than those in the methadone group (*p <* 0.001), but age at the first use of heroin and duration of treatment did not significantly differ between the groups. More participants in the buprenorphine group were satisfied with maintenance treatment than in the methadone group (85.7 ± 13.3 vs. 70.1 ± 18.3, *p <* 0.001). The buprenorphine group exhibited lower OCDUS scores than did the methadone group (*p =* 0.035), but CHQ‐12 scores did not significantly differ between the groups. Participants in the buprenorphine group had higher QoL in the physical health (*p =* 0.019), psychological health (*p =* 0.003), social interaction (*p =* 0.045), and environmental (*p =* 0.003) domains of the WHOQOL‐BREF‐T.

**TABLE 1:** Demographic data, substance use history and clinical profiles of the heroin‐dependent patients receiving maintenance treatment.

	Buprenorphine *n* = 31	Methadone *n* = 149	*p*‐Value
Demographic data			
Age (years ± SD)	47.1 ± 9.8	50.5 ± 9.7	0.079
Sex (male) (*n*, %)^a^	23 (74.2%)	126 (84.6%)	0.190
Education (years ± SD)	10.7 ± 3.2	9.4 ± 2.6	**0.013** *
<6 years^a^	5 (16.1%)	32 (21.4%)	**0.034** *
6–12 years	21 (67.7%)	112 (75.2%)	
>12 years	5 (16.1%)	5 (3.4%)	
Marriage (married) (*n*, %)^a^	14 (45.2%)	32 (21.5%)	**0.011** *
Occupation (employed) (*n*, %)^a^	28 (90.3%)	106 (70.1%)	**0.024** *
Personal income (per month) (*n*, %)			0.549
<US$ 1,000^a^	17 (54.8%)	91 (61.3%)	
US$ 1000–2000	11 (35.5%)	50 (33.3%)	
>US$ 2000	3 (9.7%)	8 (5.33%)	
Current smoker (*n*, %)^a^	31 (100.0%)	143 (96.0%)	0.592
Current alcohol drinker (*n*, %)^b^	12 (38.7%)	45 (30.0%)	0.354
Substance use history			
Age of first use heroin (years ± SD)	28.5 ± 9.3	27.3 ± 7.8	0.442
Duration of heroin use (years ± SD)	8.7 ± 7.9	14.9 ± 10.1	**<0.001** *
<10 years^b^	15 (48.4%)	44 (29.5%)	**0.041** *
≥10 years	16 (51.6%)	105 (70.5%)	
Duration of receiving treatment (years ± SD)	5.0 ± 3.9	5.4 ± 3.5	0.660
<5 years^b^	15 (48.4%)	73 (49.0%)	0.951
≥5 years	16 (51.6%)	76 (51.0%)	
Treatment satisfaction	85.7 ± 13.3	70.1 ± 18.3	**<0.001** *
*OCDUS* (score ± SD)	17.8 ± 7.6	21.3 ± 8.4	**0.035** *
*CHQ‐12* (score ± SD)	3.2 ± 1.9	4.0 ± 2.5	0.126
*WHOQOL‐BREF‐T* (score ± SD)			
Physical health	13.5 ± 2.4	12.3 ± 2.6	**0.019** *
Psychological	12.7 ± 2.8	11.1 ± 2.6	**0.003** *
Social interaction	12.9 ± 2.7	11.8 ± 2.9	**0.045** *
Environment	13.4 ± 2.6	11.7 ± 2.8	**0.003** *

* *p* < 0.05.

^**a**^ Fisher's exact test.

^**b**^ Chi‐squared test.

*p* < 0.05 is considered statistically significant.

The factors associated with QoL in univariate analysis are listed in Table 2. Compared with participants who were married, those who were unmarried exhibited lower QoL in the psychological (*p =* 0.006), social (*p =* 0.002), and environmental domains (*p =* 0.002). Compared with employment, unemployment was associated with lower QoL in the physical (*p <* 0.001), psychological (*p =* 0.002), social (*p =* 0.004), and environmental domains (*p =* 0.004). Compared with personal income of less than US$1000 per month, that of US$1000 to 2000 per month was associated with higher QoL in the physical (*p <* 0.001), psychological (*p <* 0.001), social (*p =* 0.006), and environmental domains (*p =* 0.006). Compared with nonsmokers, current smokers had higher QoL in the physical domain (*p =* 0.017). Compared with methadone treatment, buprenorphine treatment was associated with higher QoL in the physical (*p =* 0.019), psychological (*p =* 0.003), social (*p = 0*.045), and environmental domains (*p =* 0.045). The OCDUS score was negatively correlated with the physical (*p =* 0.042), social (*p =* 0.012), and environmental domains (*p =* 0.012) of QoL. The CHQ‐12 score was negatively correlated with the physical (*p <* 0.001), psychological (*p <* 0.001), social (*p <* 0.001), and environmental domains (*p <* 0.001).

**TABLE 2:** The factors associated with each domain of WHOQOL‐BREF‐T using univariate linear regression analyses.

Factor	Physical		Psychological		Social		Environmental	
	*β*	*p*	*β*	*p*	*β*	*p*	*β*	*p*
Demographic data								
Age (year)	−0.08	0.291	0	0.964	−0.03	0.709	−0.03	0.709
Sex (ref = female)	0.04	0.609	0.05	0.546	−0.05	0.488	−0.05	0.488
Education (year)	0.05	0.499	0.08	0.285	0.06	0.431	0.06	0.431
Marriage (ref = married)	−0.07	0.377	−0.21	**0.006**	−0.23	**0.002**	−0.23	**0.002**
Occupation (ref = employed)	−0.27	**<0.001**	−0.23	**0.002**	−0.21	**0.004**	−0.21	**0.004**
Personal income (ref = <US$ 1000)								
US$ 1000–2000	0.29	**<0.001**	0.29	**<0.001**	0.21	**0.006**	0.21	**0.006**
>US$ 2000	0.04	0.569	0.09	0.223	0.03	0.647	0.03	0.647
Current smoker (ref = no)	0.18	**0.017**	0.13	0.083	0.14	0.062	0.14	0.062
Current alcohol drinker (ref = no)	0.1	0.164	0.1	0.171	0.08	0.284	0.08	0.284
Substance use history								
Treatment type (ref = Methadone)	0.18	**0.019**	0.22	**0.003**	0.15	**0.045**	0.15	**0.045**
Age of first use heroin (year)	0.09	0.251	0.09	0.239	0.11	0.149	0.11	0.149
Duration of heroin use (year)	−0.14	0.068	−0.09	0.208	−0.1	0.182	−0.1	0.182
Duration of receiving treatment (year)	0.03	0.653	0.14	0.066	0.05	0.499	0.05	0.499
*OCDUS* (score)	−0.15	**0.042**	−0.14	0.062	−0.19	**0.012**	−0.19	**0.012**
*CHQ‐12* (score)	−0.45	**<0.001**	−0.41	**<0.001**	−0.37	**<0.001**	−0.37	**<0.001**

*Note*: *β* = standardized regression coefficient.

Abbreviation: ref, reference.

*p* < 0.05 is considered statistically significant.

For the four domains of QoL, we conducted multivariate linear regression using the demographic variables, drug use‐related variables, OCDUS scores, and CHQ‐12 scores as independent variables (Table 3). No collinearity existed between these independent variables. We examined the adjusted *R* ^2^ (Adj *R* ^2^) of the explanatory model for physical (Adj *R* ^2^ = 0.3143), psychological (Adj *R* ^2^ = 0.2744), social (Adj *R* ^2^ = 0.2875), and environmental (Adj *R* ^2^ = 0.1805) domains. With adjustment for other variables, unemployment was associated with lower QoL in the physical (*p =* 0.005), psychological (*p =* 0.042), and environmental domains (*p =* 0.008). Compared with personal income of less than US$1000 per month, that of US$1000 to 2000 per month was associated with higher QoL in the physical (*p =* 0.035), psychological (*p =* 0.008), and environmental domains (*p =* 0.001), and that of more than US$2000 per month was associated with higher QoL in the psychological domain (*p =* 0.048). Current smokers had higher QoL in the environmental domain (*p =* 0.038). Buprenorphine treatment (*p =* 0.032) and longer treatment duration (*p =* 0.016) were associated with higher QoL in the psychological domain.

**TABLE 3:** The factors associated with each domain of WHOQOL‐BREF‐T using multivariate linear regression analyses.

Factor	Physical		Psychological		Social		Environmental	
	Adj R‐Sq = 0.3143		Adj R‐Sq = 0.2744		Adj R‐Sq = 0.2875		Adj R‐Sq = 0.1805	
	*β*	*p*	*β*	*p*	*β*	*p*	*β*	*p*
Demographic data								
Age (year)	0.02	0.841	0.12	0.194	0.06	0.551	0.08	0.35
Sex (ref = female)	0.04	0.603	0.03	0.651	−0.06	0.439	−0.1	0.176
Education (year)	−0.03	0.67	0.02	0.772	−0.01	0.941	0.04	0.582
Marriage (ref = married)	0.04	0.564	−0.13	0.053	−0.14	0.052	−0.13	0.057
Occupation (ref = employed)	−0.2	**0.005**	−0.14	**0.042**	−0.14	0.051	−0.18	**0.008**
Personal income (ref = < US$ 1000)								
US$ 1000–2000	0.15	**0.035**	0.19	**0.008**	0.13	0.1	0.24	**0.001**
>US$ 2000	0.05	0.428	0.14	**0.048**	0.05	0.519	−0.01	0.89
Current smoker (ref = no)	0.12	0.065	0.07	0.306	0.1	0.155	0.14	**0.038**
Current alcohol drinker (ref = no)	0.08	0.217	0.08	0.22	0.08	0.261	0.02	0.792
Heroin use and treatment history								
Treatment type (ref = Methadone)	0.09	0.212	0.15	**0.032**	0.04	0.545	0.11	0.097
Age of first use heroin (year)	0.03	0.736	0.03	0.713	0.06	0.495	0.08	0.35
Duration of heroin use (year)	−0.03	0.686	0.02	0.857	0.02	0.823	0.05	0.523
Duration of receiving treatment (year)	0.07	0.323	0.16	**0.016**	0.09	0.217	0.13	0.057
*OCDUS* (score)	−0.07	0.296	−0.02	0.776	−0.09	0.238	−0.04	0.601
*Chinese Health Questionnaire* (score)	−0.38	**<0.001**	−0.33	**<0.001**	−0.28	**<0.001**	−0.31	**<0.001**

*Note*: *β* = standardized regression coefficient.

Abbreviation: ref, reference.

*p* < 0.05 is considered statistically significant.

## 4 DISCUSSION

The present study has three major findings. First, compared with the methadone group, the buprenorphine group exhibited higher treatment satisfaction, lower drug craving, and higher QoL in the physical, psychological, social, and environmental domains. Second, with adjustment for other variables, employment was positively associated with the physical, psychological, environmental domains of QoL. Third, buprenorphine treatment and longer treatment duration were associated with higher QoL in the psychological domain.

Regarding the sociodemographic characteristics, in our study, the buprenorphine group was more likely to be married, employed, and have a higher level of education. Previous studies comparing the sociodemographic characteristics of methadone and buprenorphine patients have yielded inconsistent results, possibly due to differences in drug policies. Our findings are similar to a U.S. survey that indicated that patients using buprenorphine were more likely to be employed.^38^ In contrast, in a French multicenter study, patients using methadone were more likely to have partners and stable employment.^39^ As for heroin use‐related characteristics, in our study, the methadone group had a longer history of heroin use, which may be attributed to the earlier adoption of methadone as a MOUD in Taiwan compared to buprenorphine. However, the duration of treatment did not significantly differ between the two groups. It is premature to draw conclusions from these results.

Our study indicates that the buprenorphine group had a higher treatment satisfaction compared to the methadone group. Previous studies comparing treatment satisfaction in patients treated with methadone or buprenorphine treatment have yielded inconsistent results, but overall, patients are generally satisfied with MOUD treatment.^7^ In a qualitative study, most opioid‐dependent patients expressed satisfaction with both buprenorphine and methadone treatments, with dissatisfaction primarily related to factors such as clinic visit frequency, dosing times, and the absence of take‐home doses, which may be more associated with methadone.^40^

In our study, the participants in the present study have a lower quality of life compared with healthy people. For example, the scores of WHOQOL‐BREF‐T for healthy people (defined as having no medical conditions) selected from the 2001 National Health Interview Survey are as follows: physical domain: 15.01 ± 2.10, psychological domain: 13.58 ± 2.24, social relations domain: 14.07 ± 1.96, environment domain: 13.05 ± 2.00.^31^ In another study involving elderly individuals residing in the community, after excluding respondents with psychiatric disorder, the average scores of WHOQOL‐BREF‐T were as follows: physical health 14.38 ± 3.10, psychological health 14.87 ± 3.00, social relationships 15.07 ± 2.92, environment 14.79 ± 2.46.^41^

Our study demonstrated that patients receiving buprenorphine had higher QoL than did patients receiving methadone. Because of the cross‐sectional study design, we cannot conclude that buprenorphine treatment has a more favorable effect on QoL than does methadone. The results of longitudinal studies comparing the QoL between individuals receiving methadone and buprenorphine are inconsistent, although both treatments effectively improve QoL.^15^, ^16^, ^17^, ^18^ The limitations of these studies include the open‐label design, self‐selection bias, and small sample size. It unclear whether buprenorphine is superior to methadone in improving QoL.

In our study, the buprenorphine group exhibited higher QoL in the psychological domain than did the methadone group after adjustment for other variables. Mental health problems are common among heroin users; the prevalence of depression among people with opioid use disorder ranges from 16% to 44%.^42^ The severity of psychological distress and taking medication for psychological problems are associated with lower QoL.^9^, ^43^ In particular, suicide is the leading cause of unnatural death among patients with opioid dependence in Taiwan, whereas overdose is the leading cause in the United States, emphasizing that mental health problems should be addressed among heroin users in Taiwan.^44^, ^45^

Methadone use is associated with a subjective sense of mental clouding and impaired cognitive function in several domains, possibly due to its full mu‐opioid receptor agonist activity.^46^, ^47^ From the perspective of patients with opioid dependence, treatment with methadone is associated with the fear of methadone withdrawal^48^ and methadone‐related stigmatization.^49^ Additionally, methadone is associated with a higher risk of erectile dysfunction than is buprenorphine, which is positively correlated with depression and negatively correlated with QoL.^50^, ^51^ By contrast, buprenorphine acts as a partial agonist on mu‐opioid receptors and, therefore, reduces the risk of overdose death, and the addition of naloxone further reduces its abuse potential. Previous studies suggest buprenorphine treatment effectively reduces depressive symptoms, serious suicidal ideation, and nonsuicidal self‐injury.^52^, ^53^ This antidepressant effect is assumed to be caused by the agonistic effect on mu‐opioid receptors with increases in dopamine levels, kappa receptor antagonism, and monoamine inhibition activity.^52^ Although a study did not identify different benefits for depressive symptoms between methadone and buprenorphine groups,^54^ another study reported that buprenorphine is more effective than methadone in patients with depression and dysphoria, probably because of the antagonist action on kappa opioid receptors.^55^

In this study, we demonstrated that employment is crucial in the QoL of patients receiving MOUD, which is consistent with the results of a German study.^56^ In previous studies, the lower QoL identified among heroin users may have been partly caused by these individuals being less likely to obtain or maintain a job, being prone to more frequent absenteeism or drug‐related hospitalization, and having higher rates of unemployment and lower earnings.^57^ In our study, the proportion of participants in the buprenorphine group with a job (90.3%) was significantly higher than that in the methadone group (70.1%). As take‐home methadone is not available in Taiwan, patients receiving methadone must visit the hospital every day to obtain methadone, which enhances their risk of being late to or absent from work. In contrast, buprenorphine offers the convenience of home‐based administration. In Taiwan, buprenorphine is prescribed in combination with naloxone as MOUD. Naltrexone is an opioid antagonist and is more bioavailable intravenously than sublingually. Intravenous naltrexone in heroin users induces opioid withdrawal symptoms, thereby reducing the risk of intravenous buprenorphine abuse.

In addition, our study does not exhibit a significant association between income and QoL. There has been limited research exploring the relationship between income and QoL. In the general population, even though there is a correlation between income and QoL, the correlation is very low.^58^, ^59^ The association between income and QoL may be mediated by one's attitudes towards money. For example, a study that recruited 458 American employees found no significant association between income and QoL.^60^ However, the relationship appeared to be influenced by factors such as an individual's obsession with money, job satisfaction, and demographic characteristics. The relationship between income and QoL in individuals with MOUD may be intricate and requires further research.

This study has several limitations. First, because this was a cross‐sectional study, the findings are insufficient for concluding that buprenorphine treatment more effectively improves mental health than does methadone treatment. Further longitudinal studies might be needed to address the issue. Second, some key variables that are negatively associated with QoL were not included in the explanatory model, including HIV and hepatitis C viral status and legal history. Third, patients who switched from one treatment to the other or discontinued the MOUD program were not enrolled. The difference in retention rates between the methadone group and the buprenorphine group, as well as the quality‐of‐life data for nonparticipants, could potentially influence the interpretation of the study results.

## 5 CONCLUSION

Employment was associated with higher QoL, demonstrating the importance of employment for overall wellbeing and that employment should be facilitated for patients. Longer treatment duration was associated with higher QoL, demonstrating the importance of treatment adherence. Clinicians should choose a treatment modality, such as buprenorphine or take‐home methadone, that is less likely to affect the patient's ability to maintain their job. Furthermore, we suggest that harm reduction policies may further include allowing selected patients to take methadone home or reducing the cost of buprenorphine to promote buprenorphine use.

## AUTHOR CONTRIBUTIONS

Drs. Chang and Lin conceived and designed the study. Drs. Chang, Huang and Lin recruited participants and assisted in data interpretation. Mrs. Fang performed the statistical analysis. Dr. Chang drafted the manuscript. Drs. Huang and Lin made critical revisions to the manuscript for important intellectual content.

## FUNDING INFORMATION

This research was supported by grants from the Taipei City Hospital (10601‐62‐025 and 11201‐62‐025) and the National Science and Technology Council (112‐2314‐B‐532‐003). They were not involved in the study design, data collection, analysis, and interpretation, manuscript drafting, or the decision to submit the paper for publication.

## CONFLICT OF INTEREST STATEMENT

The authors declare no conflict of interest.

## ETHICS STATEMENT

Approval of the research protocol by an Institutional Reviewer Board: This study was approved by the Ethics Committee of Taipei City Hospital (TCHIRB‐10602113) and conformed to the provisions of the Declaration of Helsinki.

Informed Consent: Yes.

Registry and the Registration No. of the study/trial: N/A.

Animal Studies: N/A.

## Data Availability

The data of the present study were not made publicly available because of the fact that the disclosure of individual data was not included in the research protocol and consent for public data sharing was not obtained from the participants. The data sets analyzed in the study are not publicly available due to ethical restrictions but are available from S.K. Lin upon reasonable request.
